# Supplementary material for: Host-microbe multiomic profiling identifies distinct COVID-19 immune dysregulation in solid organ transplant recipients
Source: Nat Commun. 2025 Jan 10;16:586. doi: 10.1038/s41467-025-55823-z (PMC11723965; doi:10.1038/s41467-025-55823-z)
Supplement: Supplementary file 1 — Supplementary Information [file 41467_2025_55823_MOESM1_ESM.pdf]

Supplementary Materials

Table of Contents

I. Supplementary Figures

Supplementary Figure 1. SARS-CoV-2 viral abundance measurements by nasal metatranscriptomics and quantitative RT-PCR are highly correlated.

Supplementary Figure 2. Days from symptom onset to hospital admission in SOT recipients and non-SOT controls were similar.

Supplementary Figure 3. Relationship between immunosuppressant administration and viral abundance, SARS-CoV-2 IgG and COVID-19 severity.

Supplementary Figure 4. Effects of SARS-CoV-2 viral abundance on differential cell type populations at Visit 1.

Supplementary Figure 5. Effects of SARS-CoV-2 viral abundance on differential protein expression at Visit 1, and on the dynamics of immune proteins.

Supplementary Figure 6. Effects of SARS-CoV-2 viral abundance on PBMC transcriptomic pathways.

Supplementary Figure 7. Effects of SARS-CoV-2 viral abundance on nasal transcriptomic pathways.

Supplementary Figure 8. Nasal gene expression is more strongly correlated with SARS-CoV-2 viral abundance than PBMC gene expression among SOT patients.

Supplementary Figure 9. Nasal biological signaling pathways associated with SARS-CoV-2 viral abundance.

Supplementary Figure 10. Principal coordinate analysis (PCoA) of Bray-Curtis dissimilarity index from nasal metatranscriptomics at Visit 1.

Supplementary Figure 11. Samples collected at each study visit for SOT recipients and non-SOT controls.

II. Supplementary Tables

Supplementary Table 1. Organ transplant type in SOT recipients.

Supplementary Table 2. Immunosuppressive treatment in SOT recipients at the time of hospital admission.

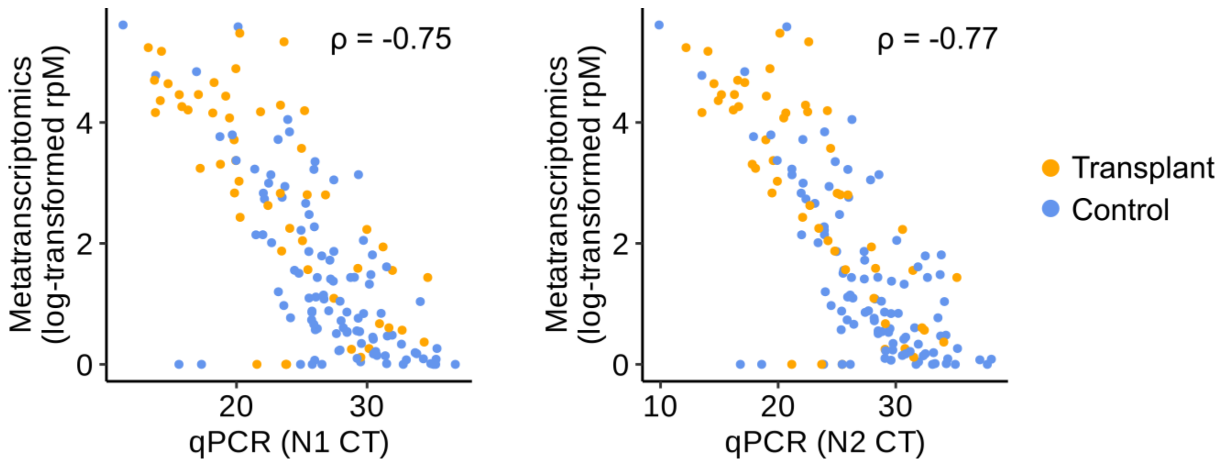

Supplementary Figure 1. SARS-CoV-2 viral abundance measurements by nasal metatranscriptomics and quantitative RT-PCR are highly correlated. Plots showing the relationship between metatranscriptomics viral reads per million (rpM) and cycle threshold (CT) value of N1 (left) and N2 (right) viral genes in transplant recipient (yellow, n=69) and controls (blue, n=137). Rho (p) indicates the Pearson's correlation coefficient.

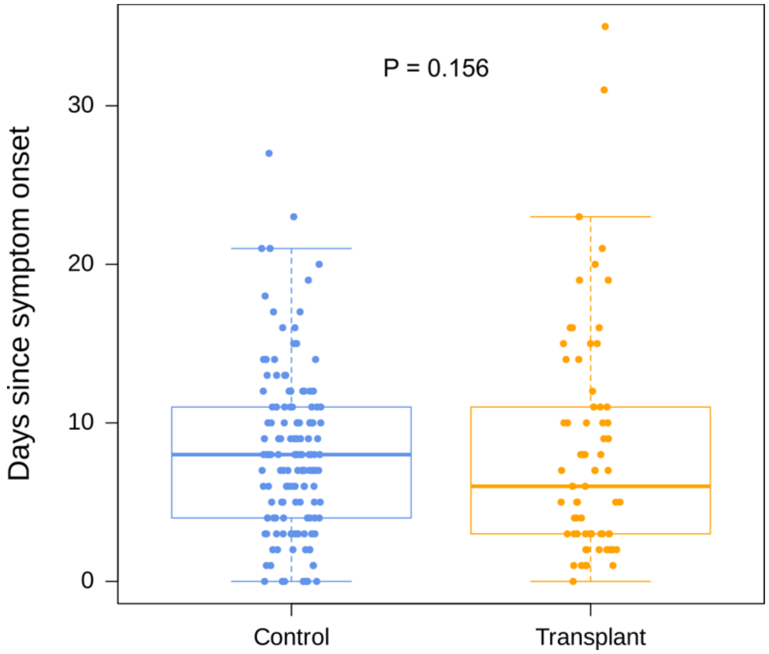

Supplementary Figure 2. Days from symptom onset to hospital admission in SOT recipients and non-SOT controls were similar. Solid organ transplant (SOT) recipients (n=72, median = 6, IQR = 3-11) versus controls (n=143, med = 8, IQR = 4-11). P-value was calculated using binomial logistic regression. Boxes show the median and interquartile range (IQR), whiskers were calculated as the 25th percentile minus 1.5 times the IQR and the 75th percentile plus 1.5 times the IQR.

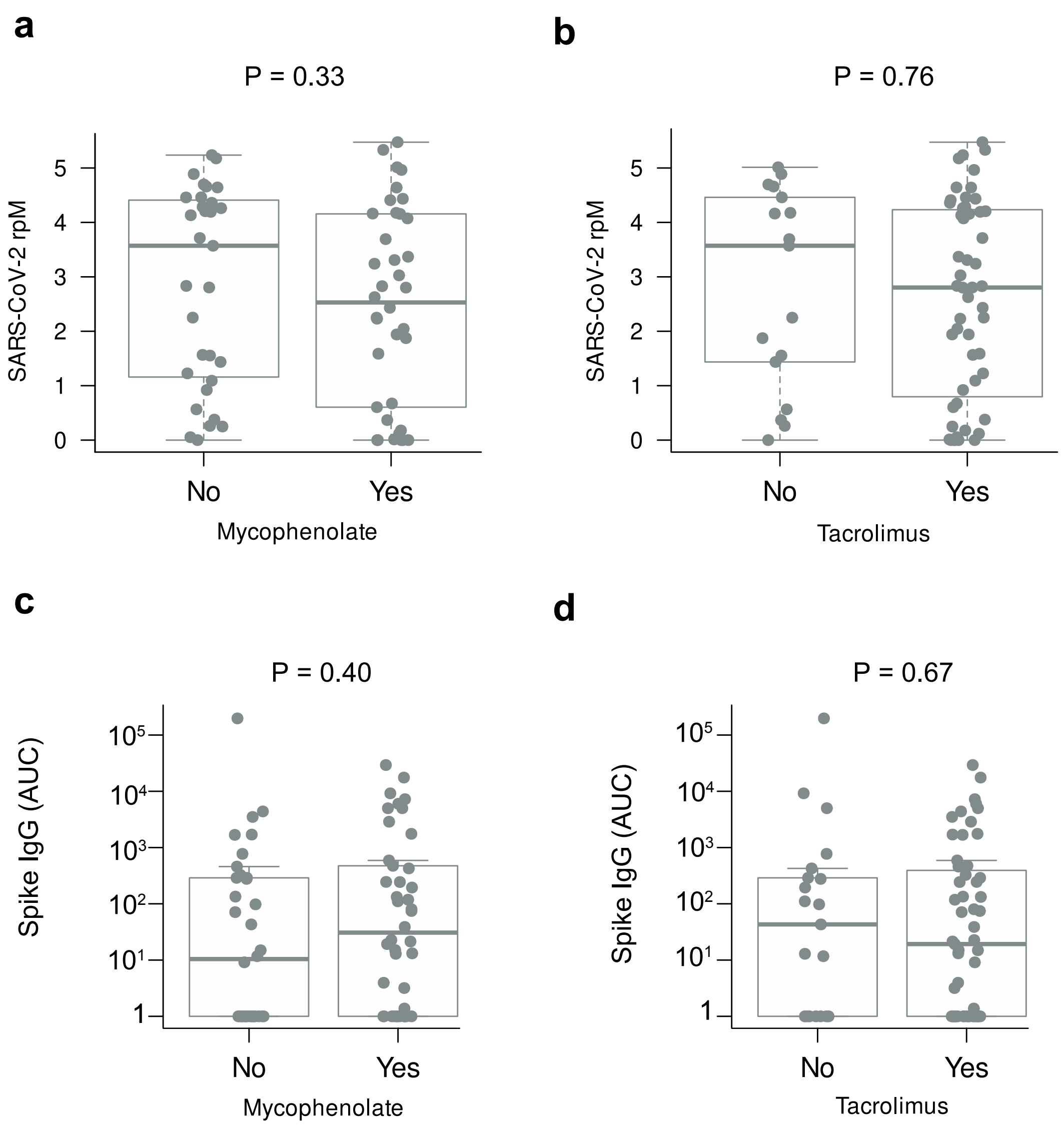

Supplementary Figure 3. Relationship between immunosuppressant administration and viral abundance, SARS-CoV-2 IgG and COVID-19 severity. (a) Relationship between mycophenolate administration (No-n=38, Yes-n=48) and fraction of patients with severe disease trajectories (IMPACC trajectory group 4 or 5). (b) Relationship between tacrolimus administration (No-n=23, Yes-n=63) and fraction of patients with severe disease trajectories (IMPACC trajectory group 4 or 5). (c) Relationship between mycophenolate administration and SARS-CoV-2 reads per million (rpM). (d) Relationship between tacrolimus administration and SARS-CoV-2 rpM. (e) Relationship between mycophenolate administration and SARS-CoV-2 spike IgG. (f) Relationship between tacrolimus administration and SARS-CoV-2 spike IgG. P-values were calculated by binomial logistic regression. Boxes show the median and interquartile range (IQR), whiskers were calculated as the 25th percentile minus 1.5 times the IQR and the 75th percentile plus 1.5 times the IQR. Abbreviations: AUC = area under the curve.

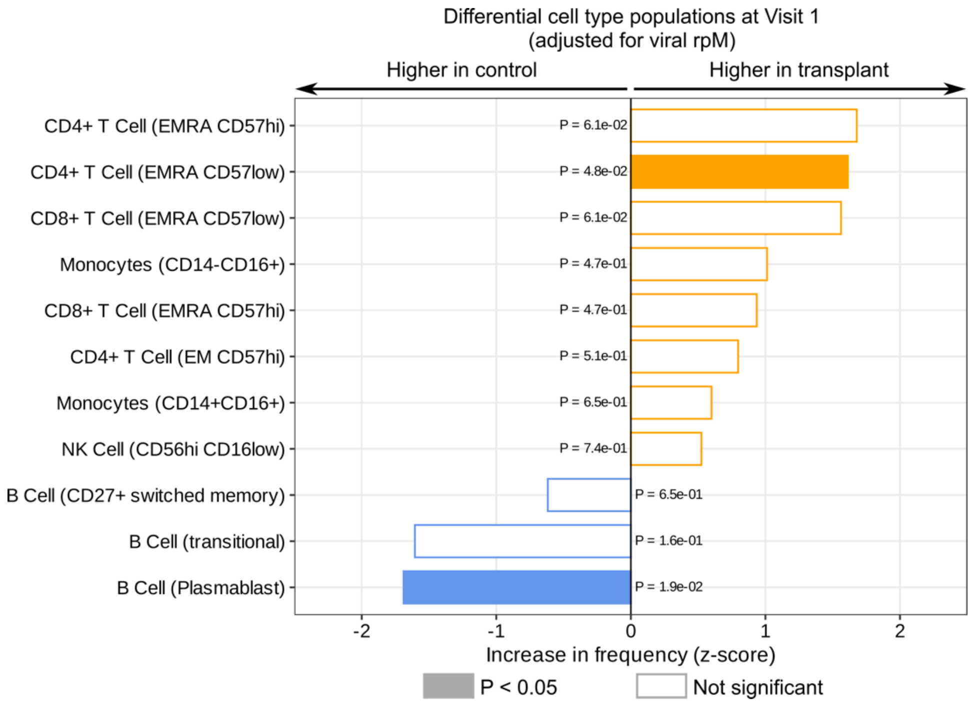

Supplementary Figure 4. Effects of SARS-CoV-2 viral abundance on differential cell type populations at Visit 1. Bar plot showing the cell types that are different between solid organ transplant (SOT, yellow, n=54) and control (blue, n=107) groups, after adjusting for SARS-CoV-2 viral reads per million (rpM). P-values were calculated using linear modelling and Benjamini-Hochberg correction. Abbreviations: EMRA = effector memory re-expressing CD45RA.

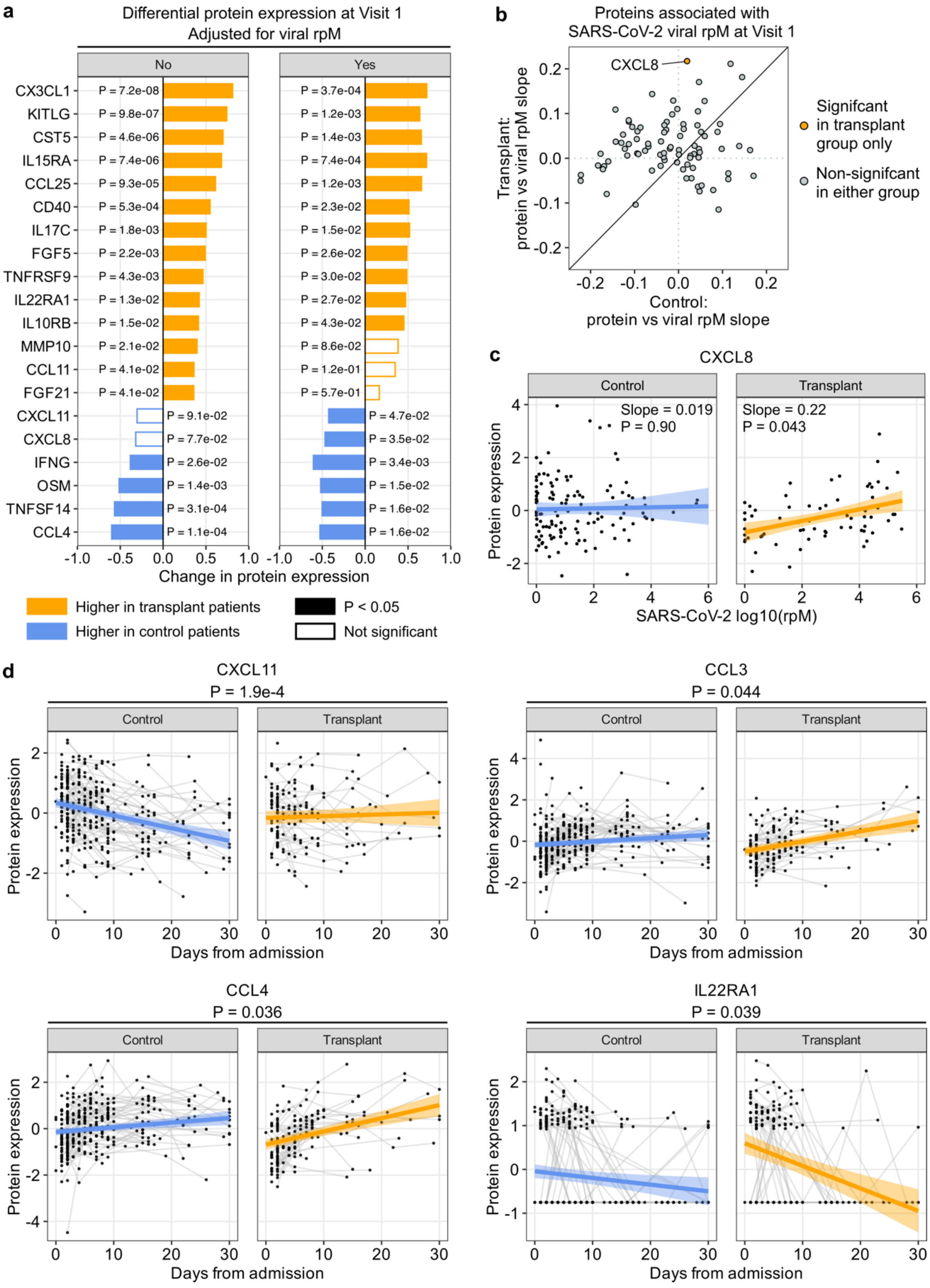
a
Differential protein expression at Visit 1
Adjusted for viral rpM
No
Yes
CX3CL1 P = 7.2e-08 P = 3.7e-04
KITLG P = 9.8e-07 P = 1.2e-03
CST5 P = 4.6e-06 P = 1.4e-03
IL15RA P = 7.4e-06 P = 7.4e-04
CCL25 P = 9.3e-05 P = 1.2e-03
CD40 P = 5.3e-04 P = 2.3e-02
IL17C P = 1.8e-03 P = 1.5e-02
FGF5 P = 2.2e-03 P = 2.6e-02
TNFRSF9 P = 4.3e-03 P = 3.0e-02
IL22RA1 P = 1.3e-02 P = 2.7e-02
IL10RB P = 1.5e-02 P = 4.3e-02
MMP10 P = 2.1e-02 P = 8.6e-02
CCL11 P = 4.1e-02 P = 1.2e-01
FGF21 P = 4.1e-02 P = 5.7e-01
CXCL11 P = 9.1e-02 P = 4.7e-02
CXCL8 P = 7.7e-02 P = 3.5e-02
IFNG P = 2.6e-02 P = 3.4e-03
OSM P = 1.4e-03 P = 1.5e-02
TNFSF14 P = 3.1e-04 P = 1.6e-02
CCL4 P = 1.1e-04 P = 1.6e-02
Change in protein expression
Higher in transplant patients
Higher in control patients
P < 0.05
Not significant
b
Proteins associated with
SARS-CoV-2 viral rpM at Visit 1
CXCL8
Transplant:
protein vs viral rpM slope
Control:
protein vs viral rpM slope
Signifcant
in transplant
group only
Non-signifcant
in either group
c
CXCL8
Control
Transplant
Slope = 0.019
P = 0.90
Slope = 0.22
P = 0.043
Protein expression
SARS-CoV-2 log10(rpM)
d
CXCL11
P = 1.9e-4
CCL3
P = 0.044
CCL4
P = 0.036
IL22RA1
P = 0.039
Control
Transplant
Protein expression
Days from admission

Supplementary Figure 5. Effects of SARS-CoV-2 viral abundance on differential protein expression at Visit 1, and on the dynamics of immune proteins. (a) Bar plots showing serum proteins that are differentially expressed between solid organ transplant (SOT) recipients (yellow, n=80) and non-SOT controls (blue, n=161) at Visit 1, with (right) and without (left) controlling for SARS-CoV-2 viral reads per million (rpM). P-values were calculated using a linear modeling and Benjamini-Hochberg correction. (b) Plot showing the slopes of serum protein expression against SARS-CoV-2 rpM. The x-axes and y-axes show the slopes in control and SOT groups, respectively. The black diagonal lines indicate y = x. (c) Plots showing CXCL8 protein expression versus viral rpM in control and SOT groups. In (b, c), P-values were calculated with linear model and Benjamini-Hochberg correction. (d) Scatter plot showing the dynamics of CXCL11, CCL3, CCL4 and IL22RA1 protein levels after hospital admission (adjusting for SARS-CoV-2 viral rpM). P-values were calculated using a linear mixed effects model and Benjamini-Hochberg correction.

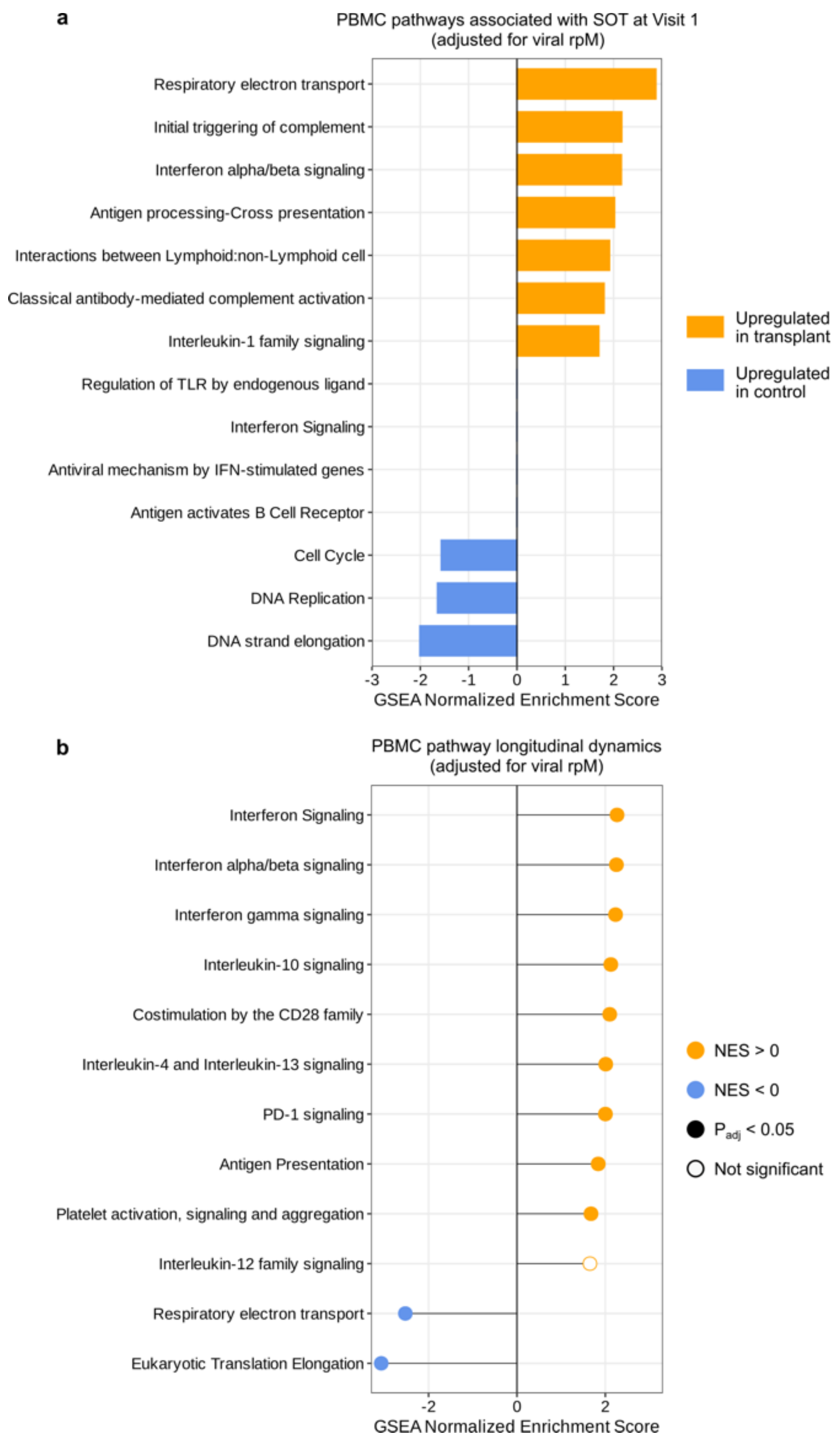

Supplementary Figure 6. Effects of SARS-CoV-2 viral abundance on PBMC transcriptomic pathways. (a) Bar plot highlighting the peripheral blood mononuclear cells (PBMC) pathways differentially enriched in solid organ transplant (SOT) recipients (yellow, n=66) versus controls (blue, n=147), with adjustment for SARS-CoV-2 viral reads per million (rpM). This plot highlights the same pathways as Figure 5b. (b) Differences in the longitudinal dynamics of PBMC signaling pathways, with adjustment for SARS-CoV-2 viral rpM. This plot highlights the same pathways as

Figure 5d. Abbreviations: GSEA = gene set enrichment analysis, NES = normalized enrichment score.

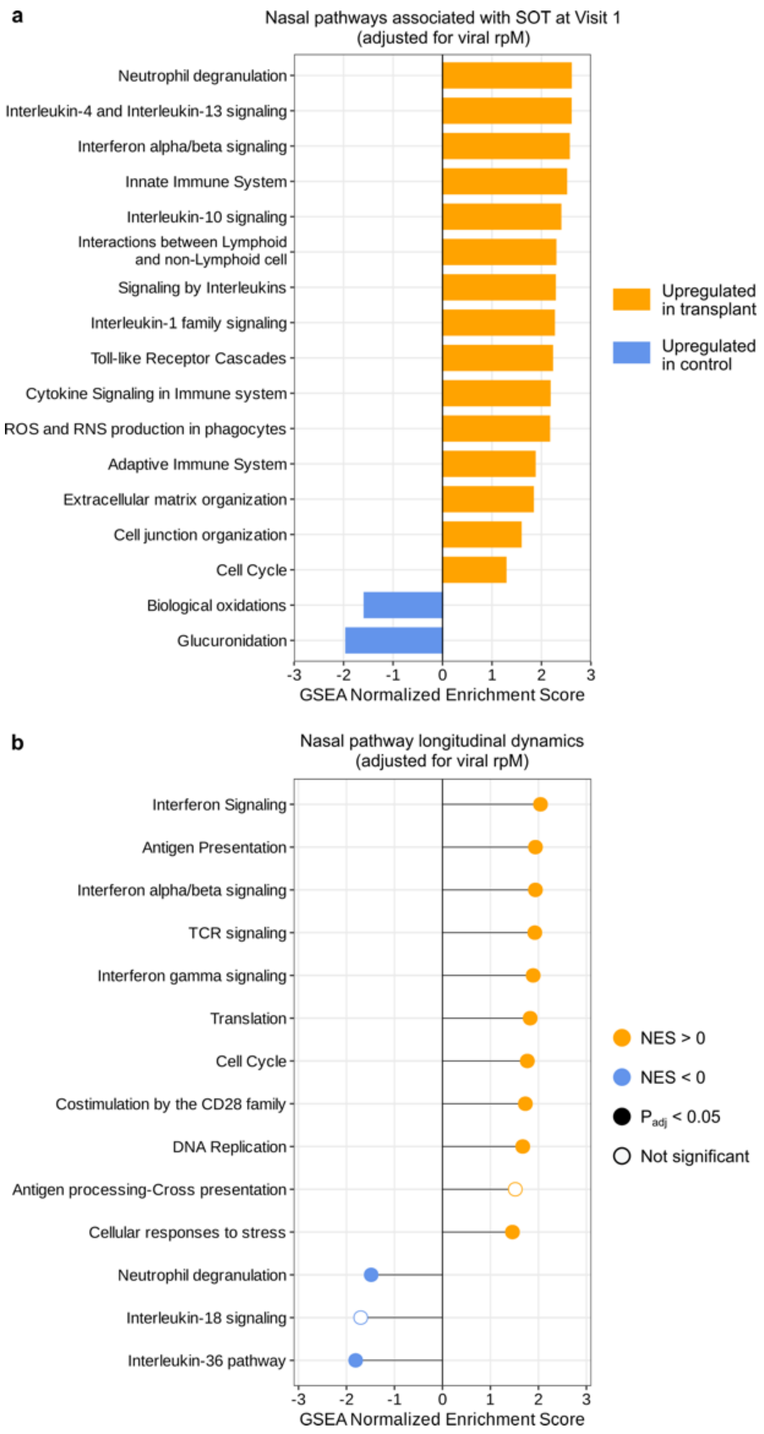

Supplementary Figure 7. Effects of SARS-CoV-2 viral abundance on nasal transcriptomic pathways. (a) Bar plot highlighting the nasal pathways differentially enriched in solid organ transplant (SOT) recipients (yellow, n=63) versus controls (blue, n=125), with adjustment for SARS-CoV-2 viral reads per million (rpM). This plot highlights the same pathways as Figure 6a. (b) Differences in the longitudinal dynamics of nasal signaling pathways, with adjustment for

SARS-CoV-2 viral rpM. This plot highlights the same pathways as Figure 6b. Abbreviations: GSEA = gene set enrichment analysis, NES = normalized enrichment score.

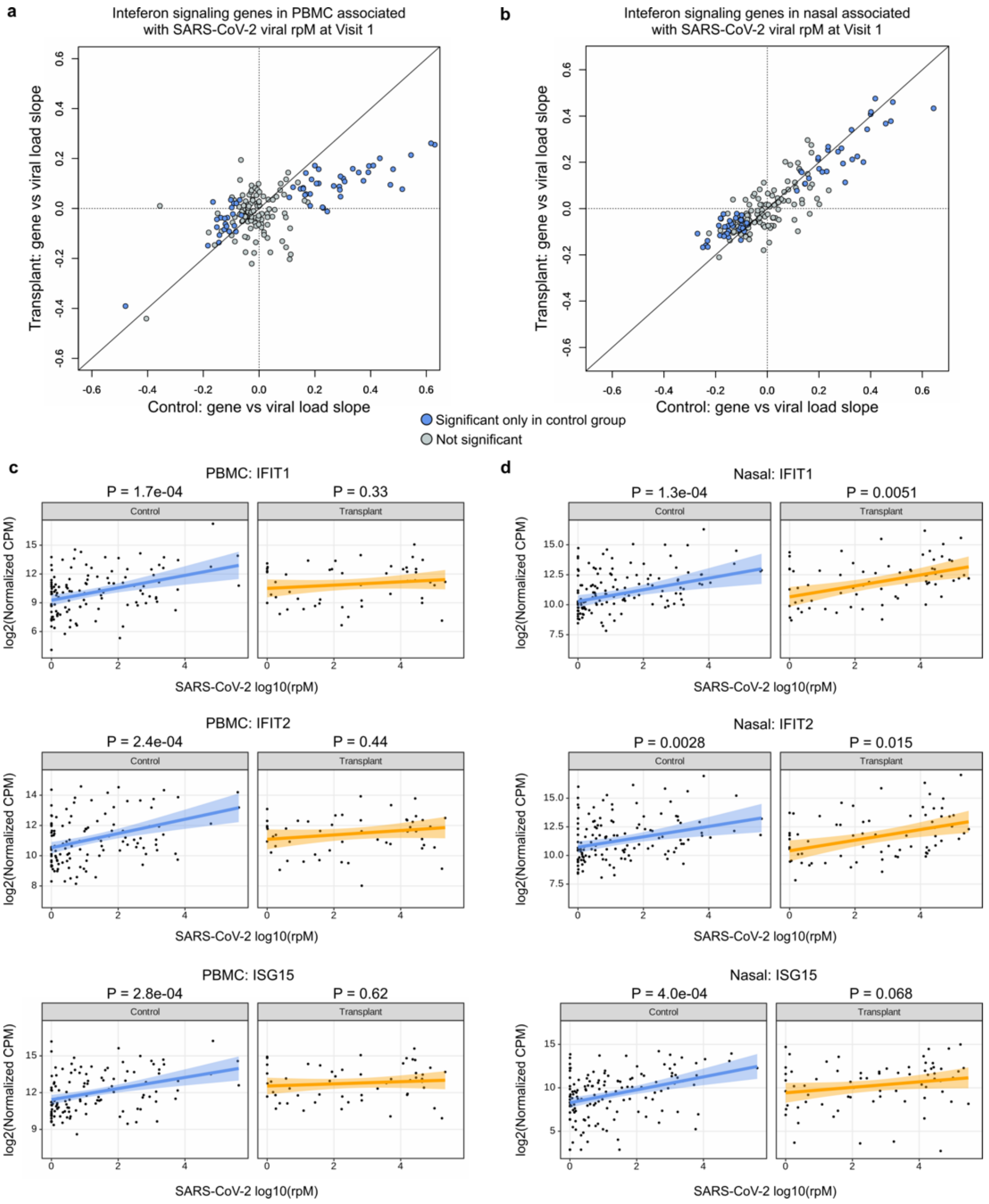

Supplementary Figure 8. Nasal gene expression is more strongly correlated with SARS-CoV-2 viral abundance than PBMC gene expression among SOT patients. (a, b) Plots showing the slopes of (a) peripheral blood mononuclear cells (PBMC) and (b) nasal gene expression against SARS-CoV-2 reads per million (rpM). The x-axes and y-axes show the slopes in control (blue, PBMC-n=147, nasal-n=125) and SOT (yellow, PBMC-n=66, nasal-n=63) groups, respectively. The black diagonal lines indicate y = x. (c, d) Plots showing the relationship of *IFIT1*, *IFIT2* and *ISG15* expression and viral rpM in PBMC and nasal samples. P-values in (a-d) were calculated using linear modeling and Benjamini-Hochberg correction.

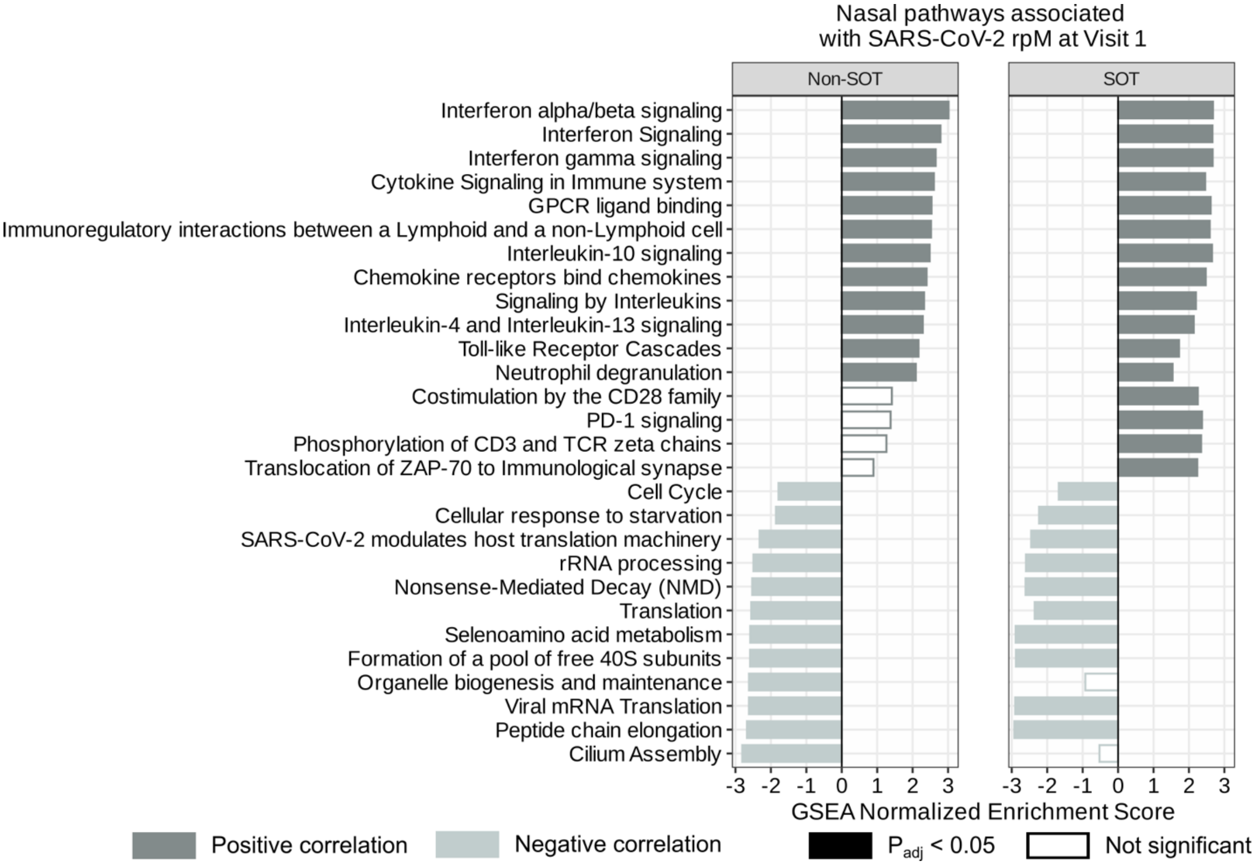

Supplementary Figure 9. Nasal biological signaling pathways associated with SARS-CoV-2 viral abundance. GSEA = gene set enrichment analysis. rpM = reads per million. Filled boxes indicate pathways demonstrating statistically significant enrichment (adjusted P < 0.05) in solid organ transplant (SOT) recipients (n=63) and non-SOT controls (n=125). P-values were calculated using a linear modeling and Benjamini-Hochberg correction.

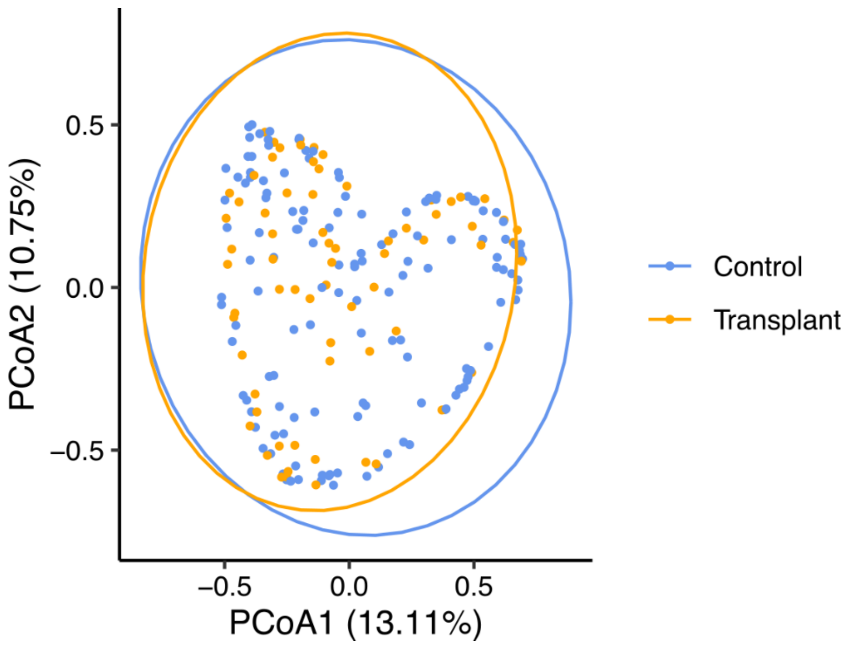

Supplementary Figure 10. Principal coordinate analysis (PCoA) of Bray-Curtis dissimilarity index from nasal metatranscriptomics at Visit 1. Colored by solid organ (transplant) recipient (yellow, n=86) versus control (blue, n=172) group with 95% confidence intervals denoted by ellipses. Significance tested with Adonis (R^2 = 0.006, P = 0.186).

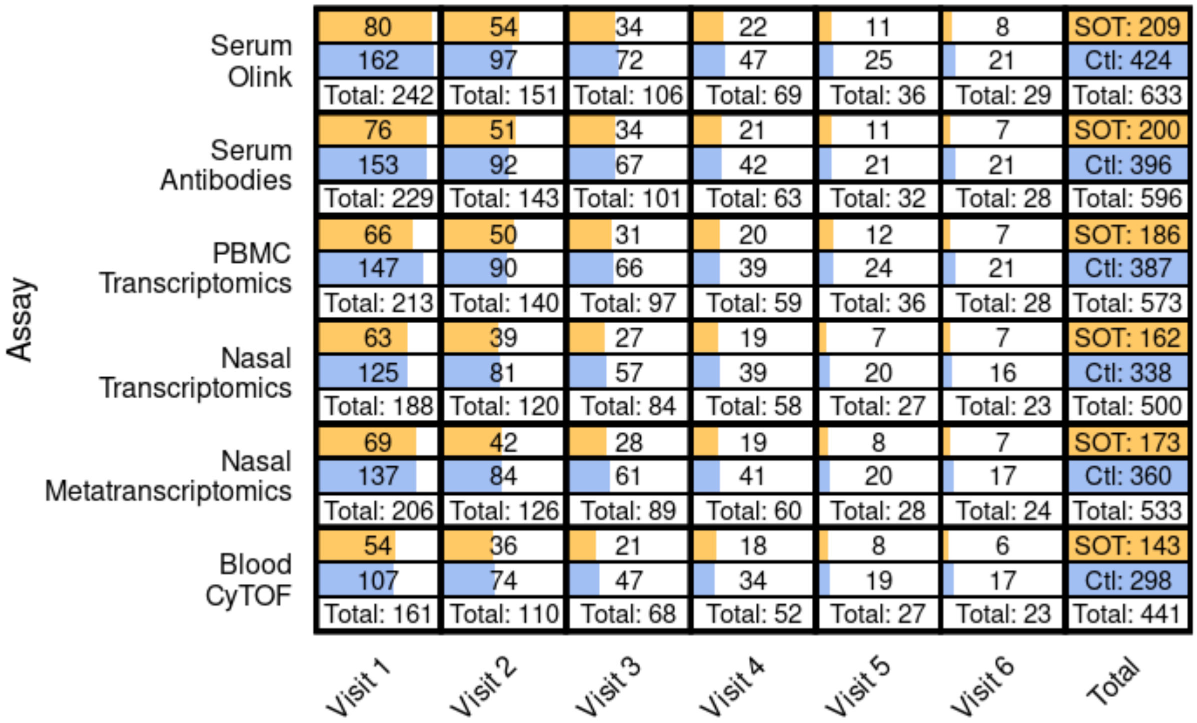

Supplementary Figure 11. Samples collected at each study visit for SOT recipients and non-SOT controls. Solid organ transplant (SOT) recipients are colored yellow and controls (Ctl) colored blue. Bar graph X axis relates to the proportion of patients in each group with available samples at each study visit. These data are provided to complement the data beneath the longitudinal panels of each main manuscript figure, which provides comparable information but instead of by study visit, by time windows following admission (number of samples between 0-10 days, 10-20 days, 20-30 days post-admission). All data points are provided in the Source Data file. Abbreviations: CyTOF = cytometry by time of flight, PBMC = peripheral blood mononuclear cells.

Supplementary Table 1. Organ transplant type in SOT recipients.

	SOT cases
**Organ type**	
Kidney	45 (52.3%)
Lung	17 (19.8%)
Liver	14 (16.3%)
Heart	10 (11.6%)

Supplementary Table 2. Immunosuppressive treatment in SOT recipients at the time of hospital admission.

Immunosuppression	Heart[1]	Kidney	Liver[2]	Lung	All
None reported	1	3	1	3	8
Azathioprine	0	0	1	0	1
Azathioprine, tacrolimus	0	1	1	0	2
Belatacept, mycophenolate	0	2	0	0	2
Cyclosporine	0	2	0	0	2
Cyclosporine, mycophenolate	0	1	0	0	1
Everolimus, tacrolimus	0	0	0	2	2
Mycophenolate	1	2	1	3	7
Mycophenolate, prednisone, tacrolimus	0	1	0	0	1
Mycophenolate, tacrolimus	6	17	7	7	37
Prednisone	0	1	1	0	2
Sirolimus, tacrolimus	1	0	0	0	1
Tacrolimus	2	11	5	2	20

[1]1/11 heart transplant recipients had also received kidney transplants.
