# Supplementary material for: Host-microbe multiomic profiling identifies distinct COVID-19 immune dysregulation in solid organ transplant recipients
Source: Nat Commun. 2025 Jan 10;16:586. doi: 10.1038/s41467-025-55823-z (PMC11723965; doi:10.1038/s41467-025-55823-z)
Supplement: Supplementary file 2 — Description of Additional Supplementary Files [file 41467_2025_55823_MOESM2_ESM.pdf]

Description of Additional Supplementary Files

Supplementary Data 1. Differentially expressed genes (FDR < 0.05) between SOT-recipients and non-SOT controls at Visit 1, based on PBMC transcriptomics.

Supplementary Data 2. Gene set enrichment analysis results based on PBMC differential gene expression (DGE) at Visit 1.

Supplementary Data 3. Longitudinal gene set enrichment analysis results based on PBMC differential gene expression between SOT-recipients and non-SOT controls.

Supplementary Data 4. Gene set enrichment analysis results based on nasal differential gene expression at Visit 1.

Supplementary Data 5. Longitudinal GSEA results based on nasal differential gene expression (DGE) between SOT-recipients and non-SOT controls.
