# Supplementary material for: Host-microbe multiomic profiling identifies distinct COVID-19 immune dysregulation in solid organ transplant recipients
Source: Nat Commun. 2025 Jan 10;16:586. doi: 10.1038/s41467-025-55823-z (PMC11723965; doi:10.1038/s41467-025-55823-z)
Supplement: Supplementary file 8 — Reporting Summary [file 41467_2025_55823_MOESM8_ESM.pdf]

nature portfolio

Corresponding author(s): Charles Langelier

Last updated by author(s): Nov 14, 2024

Reporting Summary

Nature Portfolio wishes to improve the reproducibility of the work that we publish. This form provides structure for consistency and transparency in reporting. For further information on Nature Portfolio policies, see our Editorial Policies and the Editorial Policy Checklist.

Statistics

For all statistical analyses, confirm that the following items are present in the figure legend, table legend, main text, or Methods section.

n/a	Confirmed	
☐	☒	The exact sample size (*n*) for each experimental group/condition, given as a discrete number and unit of measurement
☐	☒	A statement on whether measurements were taken from distinct samples or whether the same sample was measured repeatedly
☐	☒	The statistical test(s) used AND whether they are one- or two-sided<br>*Only common tests should be described solely by name; describe more complex techniques in the Methods section.*
☐	☒	A description of all covariates tested
☐	☒	A description of any assumptions or corrections, such as tests of normality and adjustment for multiple comparisons
☐	☒	A full description of the statistical parameters including central tendency (e.g. means) or other basic estimates (e.g. regression coefficient) AND variation (e.g. standard deviation) or associated estimates of uncertainty (e.g. confidence intervals)
☐	☒	For null hypothesis testing, the test statistic (e.g. *F*, *t*, *r*) with confidence intervals, effect sizes, degrees of freedom and *P* value noted<br>*Give P values as exact values whenever suitable.*
☒	☐	For Bayesian analysis, information on the choice of priors and Markov chain Monte Carlo settings
☒	☐	For hierarchical and complex designs, identification of the appropriate level for tests and full reporting of outcomes
☒	☐	Estimates of effect sizes (e.g. Cohen's *d*, Pearson's *r*), indicating how they were calculated

Our web collection on statistics for biologists contains articles on many of the points above.

Software and code

Policy information about availability of computer code

Data collection	All data analyses employed R v4.0.2.<br>For longitudinal analysis of SARS-CoV-2 nasal viral rpM and serum anti-Spike IgG, we used generalized additive models with mixed effects from the package gamm4 (v0.2.6) to evaluate the effects of age while controlling for sex and TG. Generalized additive modeling was preferred for these features due to their non-linear trajectories as previously reported. For all other data types, we used linear mixed effects models from the package lme4 (v1.1.25).<br>The CZ-ID pipeline was used to generate taxonomic alignments for metatranscriptomics.
Data analysis	All analysis code has been deposited at https://bitbucket.org/kleinstein/impacc-public-code/src/master/solid_organ_transplant_manuscript/.

For manuscripts utilizing custom algorithms or software that are central to the research but not yet described in published literature, software must be made available to editors and reviewers. We strongly encourage code deposition in a community repository (e.g. GitHub). See the Nature Portfolio guidelines for submitting code & software for further information.

Data

Policy information about availability of data

All manuscripts must include a data availability statement. This statement should provide the following information, where applicable:

- Accession codes, unique identifiers, or web links for publicly available datasets
- A description of any restrictions on data availability
- For clinical datasets or third party data, please ensure that the statement adheres to our policy

Data files are available at ImmPort under accession number SDY1760 and dbGAP accession number phs002686.v1.p1. All analysis code has been deposited at https://bitbucket.org/kleinstein/impacc-public-code/src/master/solid_organ_transplant_manuscript/.

Research involving human participants, their data, or biological material

Policy information about studies with human participants or human data. See also policy information about sex, gender (identity/presentation), and sexual orientation and race, ethnicity and racism.

Reporting on sex and gender	Findings apply to both sexes. All analyses were adjusted for sex, considering it as a biological variable.
Reporting on race, ethnicity, or other socially relevant groupings	Self-reported race and ethnicity were compared between groups in this study, including the following categories:<br>Ethnicity:<br>Hispanic or Latino<br>Not Hispanic or Latino<br>Not Specified<br>Race:<br>American Indian/Alaska Native<br>Asian<br>Black/African American<br>Multiple<br>Other/Declined<br>Unknown/Unavailable<br>White
Population characteristics	Population characteristics are provided in Table 1.
Recruitment	This study leveraged data from the IMPACC cohort18,19, which enrolled 1286 participants from 20 hospitals across 15 medical centers in the United States between May 5th, 2020 and March 19th, 2021. Eligible participants were participants hospitalized with SARS-CoV-2 infection confirmed by RT-PCR and symptoms or signs consistent with COVID-19. Solid organ transplant (SOT) patients were identified by review of medication list for immunosuppressive medications. Patients identified as SOT recipients were confirmed by chart review to verify transplant status and organ type.
Ethics oversight	NIAID staff conferred with the Department of Health and Human Services Office for Human Research Protections (OHRP) regarding potential applicability of the public health surveillance exception [45CFR46.102 (l) (2)] to the IMPACC study protocol. OHRP concurred that the study satisfied criteria for the public health surveillance exception, and the IMPACC study team sent the study protocol, and participant information sheet for review, and assessment to institutional review boards (IRBs) at participating institutions. Twelve institutions elected to conduct the study as public health surveillance, while three sites with prior IRB-approved biobanking protocols elected to integrate and conduct IMPACC under their institutional protocols (University of Texas at Austin, IRB 2020-04-0117; University of California San Francisco, IRB 20-30497; Case Western reserve university, IRB STUDY20200573) with informed consent requirements. Participants enrolled under the public health surveillance exclusion were provided information sheets describing the study, samples to be collected, and plans for data de-identification, and use. Those that requested not to participate after reviewing the information sheet were not enrolled. Participants did not receive compensation for study participation while hospitalized, and subsequently were offered compensation during outpatient follow-up.

Note that full information on the approval of the study protocol must also be provided in the manuscript.

Field-specific reporting

Please select the one below that is the best fit for your research. If you are not sure, read the appropriate sections before making your selection.

☒ Life sciences ☐ Behavioural & social sciences ☐ Ecological, evolutionary & environmental sciences

For a reference copy of the document with all sections, see nature.com/documents/nr-reporting-summary-flat.pdf

Life sciences study design

All studies must disclose on these points even when the disclosure is negative.

Sample size	We conducted a case-control study of patients within the IMPACC cohort (n=1286), matching all 86 solid organ transplant (SOT) recipients in

	the cohort 2:1 by age, sex and study site with 172 immunocompetent controls.
Data exclusions	For the O-link proteomics analysis we excluded proteins that were detected in fewer than 20% of samples, resulting in 84 proteins for analysis.
Replication	This case control study was done in a single, multicenter cohort. No other cohorts with comparable biological data and annotation of solid organ transplantation status are available for replication.
Randomization	This is not a clinical trial so patients were not randomized to an intervention.
Blinding	This was not a clinical trial so blinding was not necessary.

Reporting for specific materials, systems and methods

We require information from authors about some types of materials, experimental systems and methods used in many studies. Here, indicate whether each material, system or method listed is relevant to your study. If you are not sure if a list item applies to your research, read the appropriate section before selecting a response.

Materials & experimental systems

n/a	Involved in the study
☒	☐ Antibodies
☒	☐ Eukaryotic cell lines
☒	☐ Palaeontology and archaeology
☒	☐ Animals and other organisms
☐	☒ Clinical data
☒	☐ Dual use research of concern
☒	☐ Plants

Methods

n/a	Involved in the study
☒	☐ ChIP-seq
☒	☐ Flow cytometry
☒	☐ MRI-based neuroimaging

Clinical data

Policy information about clinical studies

All manuscripts should comply with the ICMJEguidelines for publication of clinical research and a completedCONSORT checklist must be included with all submissions.

Clinical trial registration	Not a clinical trial.
Study protocol	The IMPACC cohort study protocol is described in detail in:<br>1. Arce, Joann et al. Multi-omic longitudinal study reveals immune correlates of clinical course among hospitalized COVID-19 patients. Cell Reports Medicine (2023).<br>2. IMPACC Manuscript Writing Team & IMPACC Network Steering Committee. Immunophenotyping assessment in a COVID-19 cohort (IMPACC): A prospective longitudinal study. Sci Immunol 6, eabf3733 (2021).
Data collection	This study leveraged data from the IMPACC cohort18,19, which enrolled 1286 participants from 20 hospitals across 15 medical centers in the United States between May 5th, 2020 and March 19th, 2021.
Outcomes	Outcomes were measurements of gene expression, protein levels, cell frequencies and microbial abundances, which were compared between solid organ transplant recipients and controls.

Plants

Seed stocks	*Report on the source of all seed stocks or other plant material used. If applicable, state the seed stock centre and catalogue number. If plant specimens were collected from the field, describe the collection location, date and sampling procedures.*
Novel plant genotypes	*Describe the methods by which all novel plant genotypes were produced. This includes those generated by transgenic approaches, gene editing, chemical/radiation-based mutagenesis and hybridization. For transgenic lines, describe the transformation method, the number of independent lines analyzed and the generation upon which experiments were performed. For gene-edited lines, describe the editor used, the endogenous sequence targeted for editing, the targeting guide RNA sequence (if applicable) and how the editor was applied.*
Authentication	*Describe any authentication procedures for each seed stock used or novel genotype generated. Describe any experiments used to assess the effect of a mutation and, where applicable, how potential secondary effects (e.g. second site T-DNA insertions, mosiacism, off-target gene editing) were examined.*
